# Evaluation of a long‐acting injectable formulation of omeprazole in healthy dogs

**DOI:** 10.1111/jvim.16440

**Published:** 2022-05-12

**Authors:** Adesola Odunayo, Gina Galyon, Joshua Price, Silke Hecht, M. Katherine Tolbert

**Affiliations:** ^1^ Department of Small Animal Clinical Sciences University of Florida Gainesville Florida USA; ^2^ Department of Small Animal Clinical Sciences University of Tennessee Knoxville Tennessee USA; ^3^ Office of Information and Technology University of Tennessee Knoxville Tennessee USA; ^4^ Gastrointestinal Laboratory, Department of Small Animal Clinical Sciences Texas A&M University College Station Texas USA

**Keywords:** acid suppression, bravo monitoring, canine intragastric pH, proton pump inhibitor

## Abstract

**Background:**

To evaluate the efficacy of a single intramuscular adminsitration of long‐acting omeprazole (LA‐OMEP) in increasing gastric pH in dogs.

**Hypothesis:**

We hypothesized that LA‐OMEP would meet in healthy dogs the clinical goals defined for human patients for treatment of gastroduodenal ulceration.

**Animals:**

Nine healthy research dogs.

**Methods:**

Prospective experimental study. Dogs were given a 4 mg/kg intramuscular injection of LA‐OMEP. Intragastric pH was continuously recorded on treatment days 0 to 7. Daily mean pH and mean percentage time (MPT) intragastric pH was ≥3 or ≥4 were determined.

**Results:**

The mean onset of action for the LA‐OMEP was 98.11 min (SD 46.39). The mean number of days the dogs' pH met established goals for MPT pH ≥3 was 5.5 days (range, 3‐7) and 5.25 days for MPT pH ≥4 (range, 3‐7). Long‐acting omeprazole met the human clinical goals pH ≥3 for 72 hours in 8/8 of the dogs and MPT pH ≥4 for 96 hours in 7/8 of dogs.

**Conclusions and Clinical Importance:**

The LA‐OMEP formulation produced gastric acid suppression in healthy dogs for an average of 5 days and up to 7 days, after a single intramuscular injection. No major adverse effects were observed.

**Abbreviations**
GI: gastrointestinal
LA‐OMEP: long‐acting injectable omeprazole
MPT: mean percentage time
PPI: proton pump inhibitor
RAH: rebound acid hypersecretion

## 1 INTRODUCTION

Gastrointestinal (GI) bleeding is common in critically ill dogs and is a cause of increased morbidity and case fatality.^1^, ^2^ Clinical signs of GI bleeding include anorexia, lethargy, vomiting, abdominal pain, hematemesis, melena, and hematochezia.^3^, ^4^ Central to the treatment of upper GI bleeding is inhibition of gastric acid suppression through the administration of gastric acid‐suppressant treatment.^1^, ^4^, ^5^

In human patients, the use of proton pump inhibitors (PPIs) reduces both bleeding risk and the need for endoscopic intervention in bleeding patients.^4^, ^6^ Suppressing gastric acid production and maintaining intragastric pH ≥3 and ≥4 for 75% and 67% of the day promotes the healing of duodenal ulcers and gastroesophageal reflux disease in human patients, respectively.^7^, ^8^

In dogs, PO and IV administation of PPIs are superior to standard doses of histamine‐2 receptor antagonists in increasing intragastric pH for treating gastric/duodenal ulceration as well as moderate to severe esophagitis.^9^, ^10^ However, although there are no established clinical goals for acid suppression in dogs, IV administration of pantoprazole to dogs for treatment of duodenal ulceration does not consistently meet the aforementioned clinical goals established for humans.^10^, ^11^

Recently, long‐acting injectable omeprazole (LA‐OMEP) was evaluated in horses (100 mg/mL, Luoda Pharma, Caringbah, NSW, Australia).^12^, ^13^ This novel omeprazole product, suspended in a vehicle selected for tissue tolerance, provided acid suppression in horses after a single injection for up to 7 days. The single injection met the clinical goals for acid suppression defined in people in 57% (4/7) of horses for all 7 days and 100% (7/7) of horses for 4 days.^12^ A potential advantage of this formulation in dogs would be sustained acid suppression after a single injection and increased treatment compliance for dogs with severe upper GI bleeding as missing even 1 dose of an oral PPI could have deleterious effects on maintaining desired acid suppression.^14^ The LA‐OMEP formulation could also potentially reduce the duration of hospitalization in affected dogs.

The study objective was to evaluate the efficacy of LA‐OMEP in increasing intragastric pH in dogs after a single intramuscular injection. We hypothesized that LA‐OMEP would meet the clinical goals defined for human patients in our cohort of healthy dogs.

## 2 MATERIALS AND METHODS

### 2.1 Study animals

The study protocol was approved by the Institutional Animal Care and Use Committee at the University of Tennessee. Nine adult healthy purpose‐bred Beagle dogs from a research colony at the University of Tennessee were enrolled in the study (4 spayed females, 5 castrated males), all approximately 5 years of age and weighing 9 to 14.1 kg (median 12.3 kg).

The number of dogs was selected based on a sample size calculation using the study that evaluated LA‐OMEP in horses.^12^ In order to detect a 20% change in mean percentage time (MPT) pH is ≥4, using a conservative SD of 16.07 and assuming a moderate correlation of 0.6 and an alpha of 0.05, 7 dogs were needed to have an 80% power of finding significant differences over time, if they existed. Two additional dogs were enrolled to account for potential study dropout.

All dogs were deemed healthy based on a physical exam performed at the beginning of the study and recent diagnostic tests (CBC, serum biochemistry, urinalysis, and a fecal examination). The dogs were maintained in a closed colony and received monthly preventative care, including an anthelmintic. In order to detect any adverse effects from LA‐OMEP, the dogs were monitored for inappetence (characterized by consuming <50% of their meals on more than 3 consecutive occasions), weight loss >10% of their body mass, >3 episodes of vomiting in a 24‐hour period or diarrhea characterized by a Purina fecal score >/=5 for more than a 48‐hour period. The dogs were fed their normal commercial dry food diet, Purina One Smart Blend Lamb and Rice Formula (Nestlé Purina PetCare Company, St. Louis, Missouri) once daily in the morning and water was given ad libitum throughout the study. The injection site was also monitored 3 times a day for adverse effects and was graded using a modification of a previously published grading system evaluating for pain, tenderness, swelling, necrosis, and ulceration.^15^

### 2.2 Intragastric pH capsule placement

On the morning of day 0, the morning meal was withheld and the dogs were sedated using butorphanol (0.2 mg/kg IV; Torbugesic 10 mg/mL injection; Fort Dodge Animal Health, FortDodge, Iowa) and dexmedetomidine (5‐10 μg/kg IV; Dexdomitor 0.5 mg/mL injection; Orion Pharma, Espoo, Finland). The Bravo pH capsules (Medtronic, Minneapolis, Minnesota) were placed utilizing digital radiology for assistance. Intragastric pH capsules were adhered to the dogs' gastric mucosa as described in other studies.^11^ After the procedure, the sedation was reversed with an equal volume of atipamezole (0.05‐0.1 mg/kg IM; Antisedan 5 mg/mL injection; Orion Pharma).

### 2.3 Study design

A prospective, experimental nonrandomized study was designed. After the pH capsule was placed on the morning of day 0, baseline intragastric pH data were collected the rest of that day. On day 1, all dogs received 4 mg/kg of the 100 mg/mL LA‐OMEP formulation (0.36‐0.56 mL, median 0.49 mL), stored and administered according to the manufacturer's recommendations, intramuscularly (IM) in the right lumbar epaxial muscle. Adverse effects including general attitude, number of vomiting episodes, number of daily defecations, injection site reactions, and fecal score were recorded every 8 hours. Fecal scores were graded using a standardized fecal scoring system (Fecal Scoring System, Nestlé Purina PetCare Company). A CBC and serum biochemistry were obtained 2 weeks after conclusion of the study.

### 2.4 Intragastric pH monitoring

Intragastric pH was recorded continuously for at least 8 days after capsule placement starting on day 0 and continuing through day 7 or until the capsule detached, if detachment occurred before day 7. The Bravo pH capsule naturally detaches from the gastric mucosa within 2 to 4 days after placement. A new capsule was placed in each dog on day 3, and on any day capsule detachment occurred before the end of day 7. Intragastric pH was continued to be monitored if the pH capsule remained attached to the gastric mucosa after day 7. Telemetric data from the capsules were transferred to a corresponding recorder that was placed within 3 feet or 1 m of the dog and remained with the dog throughout the observation period. pH data were uploaded to a commercial computer software system (Reflux Software v6.1, Medtronic) every 24 hours. The receiver was then reset after the data were uploaded, and the same receiver was used to capture the next 24 hours of data. A right lateral radiograph was taken to confirm the pH capsule was still within the stomach if early gastric detachment and passage was suspected based on a rapid and sustained increase in the intragastric pH >4. The onset of action of LA‐OMEP in each dog was determined by the time after which the intragastric pH was persistently >4 for at least 60 minutes.

### 2.5 Statistical analysis

A single factor repeated measures mixed‐model ANOVA was performed to evaluate mean pH, MPT pH ≥3, and MPT pH ≥4 for difference over time. Cohort and dog nested within cohort were considered random effects in each analysis. Tukey‐Kramer post hoc test *P*‐value adjustments were performed to control for type 1 errors. A Shapiro‐Wilk test and QQ plots were used to evaluate normality of ANOVA residuals for each outcome. Levene's equality of variances test was used to evaluate equality of variances between days. Box‐and‐Whisker plots and studentized residual diagnostics were performed to evaluate each mixed model for the presence of outliers. All statistical assumptions regarding normality and equality of variances were met. Statistical significance was defined as *P* < .05. Statistical analysis was performed using commercial software (SAS software, version 9.4, Cary, North Carolina; Release TS1M7). Figures were created using commercially available software (Prism8, GraphPad Software, San Diego, California).

## 3 RESULTS

### 3.1 pH capsule placement

A total of 42 capsules were deployed during the study. Eleven capsules failed and required re‐deployment on day 0 (n = 4), day 3 (n = 4), day 4 (n = 1), day 5 (n = 1), and day 7 (n = 1) of the study. One dog had only 1 capsule placed and was terminated from the study due to a seizure (see adverse effects). Two dogs required 3 capsules for the study duration and 6 dogs required 4 capsules for the study duration. When capsule displacement occurred, the data available until the time of detachment were retained for analysis. Most capsules were placed on days 0, 3, and 7. All capsules were replaced between 44 minutes to 8 hours and 33 minutes of pH capsule detachment from the gastric mucosa.

### 3.2 Intragastric pH recording

**FIGURE 1:**
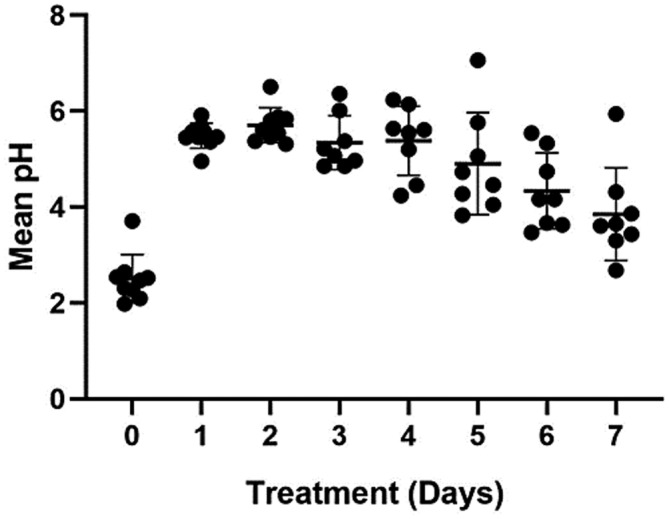
The mean intragastric pH on treatment days 1 to 7 for all dogs receiving a single dose of 4 mg/kg long‐acting omeprazole intramuscularly. The shaded dot and horizontal and vertical lines represent the individual dog mean pH, and group mean and standard deviations, respectively. Significant difference in mean pH was observed between days 0 and all other days (*P* ≤ .001 for each)

**FIGURE 2:**
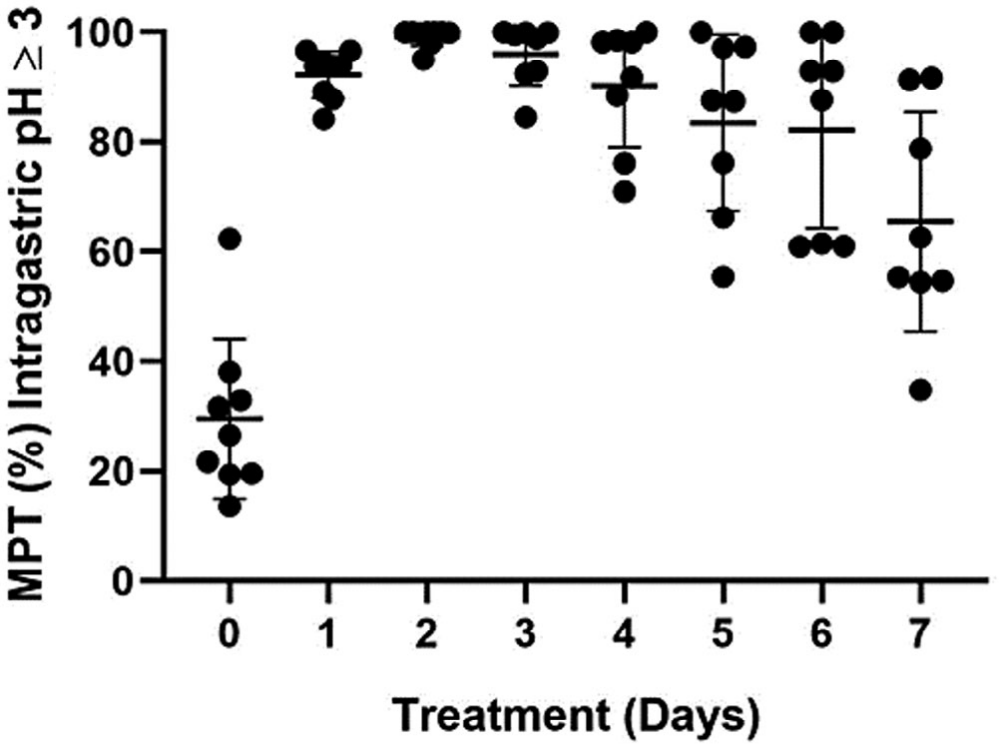
The mean percentage time (MPT) intragastric pH ≥3 on treatment days 1 to 7 for all dogs receiving a single dose of 4 mg/kg long‐acting omeprazole intramuscularly. The shaded dot and horizontal and vertical lines represent the individual dog mean pH, group mean, and standard deviations, respectively. Significant differences in MPT intragastric pH ≥3 were noted between days 0 and all other days (*P* ≤ .001 for each)

**FIGURE 3:**
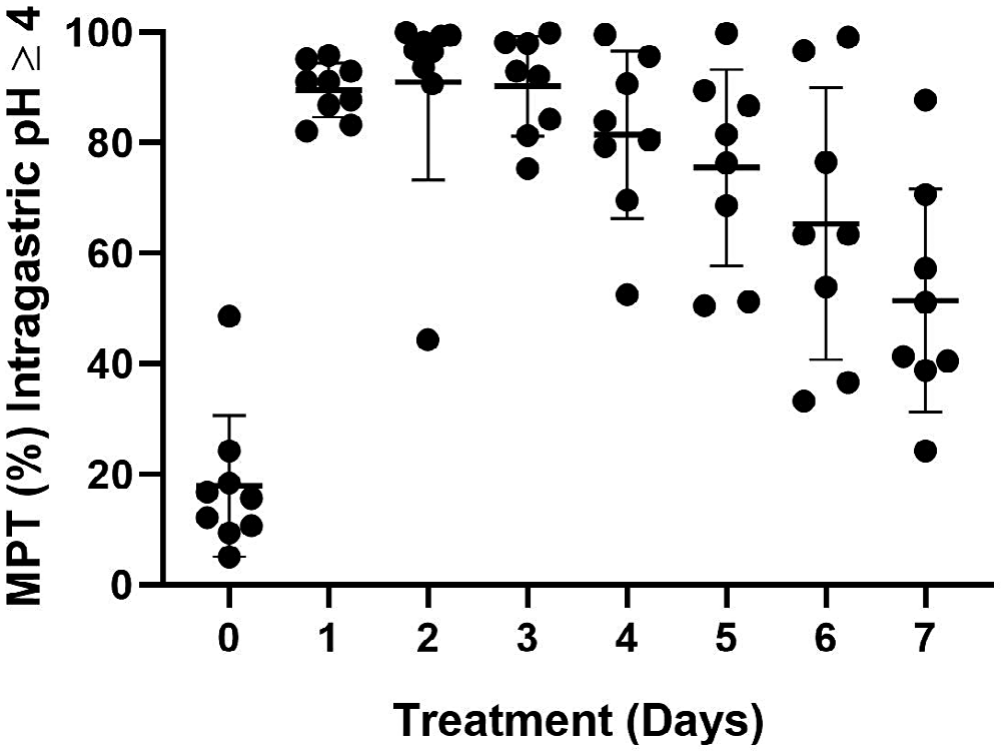
The mean percentage time (MPT) intragastric pH ≥4 on treatment days 1 to 7 for all dogs receiving a single dose of 4 mg/kg long‐acting omeprazole intramuscularly. The shaded dot, horizontal and vertical lines represent the individual dog mean, group mean and standard deviations, respectively. Significant increases in MPT intragastric pH ≥4 were noted between days 0 and all other days (*P* ≤ 0.001 for each)

The mean intragastric pH and the MPT intragastric pH were ≥3 and ≥4 are graphically depicted in Figures 1, 2, 3, respectively.

Intragastric pH on day 0 was significantly lower than all days including day 7 (*P* < .001, for each). There was no difference observed between days 1 and 5. Intragastric pH on day 6 was significantly lower than days 1 to 4 (*P* ≤ .03, for each) but did not differ from day 5. Intragastric pH on day 7 was significantly lower than days 1 to 5 (*P* ≤ .02, for each) but did not differ from day 6.

When evaluating MPT pH ≥3, an overall mean difference was observed between days (*P* < .001). Intragastric pH on day 0 was significantly lower than all other days, including day 7 (*P* < .001, for each). Intragastric pH on day 7 was significantly lower than days 1 to 4 (*P* ≤ .002, for each) but did not differ from days 5 or 6. No difference was observed between days 1 to 6 or days 5 to 7.

For MPT pH ≥4, an overall mean difference was observed between days (*P* < .001). Intragastric pH on day 0 was significantly lower than all other days, including day 7 (*P* ≤ .001, for each). Intragastric pH on day 6 was significantly lower than days 1 to 3 (*P* ≤ .04, for each) but did not differ from days 4 to 5. Intragastric pH on day 7 was significantly lower than days 1 to 5 (*P* ≤ .05, for each) but did not differ from day 6.

The mean number of days the dogs' pH met established goals for MPT pH ≥3 was 5.5 days and 5.25 days for MPT pH ≥4. Four dogs had pH recordings captured after day 7. These data were not included in the statistical review due to the small amount of data collected.

### 3.3 Onset of action

The mean onset of action for the LA‐OMEP in all 9 dogs was 98.11 minutes (SD 46.39 minutes). The most rapid onset of action was 45 minutes in 1 dog and the slowest onset of action was 183 minutes.

## 4 ADVERSE EFFECTS

Eight dogs completed the study. One female dog was removed from the study due to a single seizure. That dog had a grand mal seizure on day 1, about an hour after the LA‐OMEP was administered. A physical and neurologic examination (performed by the neurology service) did not detect abnormalities. Serum electrolyte and blood glucose concentration were all within the normal reference range. The dog was monitored overnight and did not have any additional seizures. The pH data from that dog were captured through day 3 until the pH capsule detached from the gastric mucosa. The dog was excluded from completing the study beyond this point.

There were 3 episodes of vomiting by 3 dogs. These vomiting episodes happened on days 1, 6, and 7. Two of the vomiting episodes happened on days 6 and 7, after the dogs were recovering from the pH capsule placement. There were 4 episodes of hyporexia where <50% of the food was consumed by 3 dogs. All the episodes of hyporexia were associated with sedation on pH capsule placement days. The median fecal score was 2.5 (1‐4.6). There were 7 episodes, from 6 dogs, where the fecal score was >/=5. All episodes where the fecal score was >/=5 were associated with sedation on pH capsule placement days.

There were no injection site adverse effects noted in any dog on any day. No dog lost >10% of their baseline bodyweight during the study. Results from a complete blood count and serum biochemistry panel obtained 2 weeks after the study did not reveal any abnormalities.

## 5 DISCUSSION

A single 4 mg/kg intramuscular administration of the LA‐OMEP resulted in sustained pH suppression in healthy dogs. Although the individual response of each dog to the LA‐OMEP varied, on average, gastric acid suppression was achieved continuously for about 5 days. In addition, in all dogs, the mean pH had not returned to baseline pH by day 7, showing that LA‐OMEP was still suppressing gastric acid secretion even though it did not meet established human clinical goals for the treatment of acid‐related disorders.

The efficacy and duration of action of LA‐OMEP in this group of healthy dogs has the potential advantages of rapid onset and sustained continuous gastric acid suppression, reduced duration of hospitalization, reduced cost of hospitalization, and improved treatment compliance. Although these potential benefits still need to be investigated in clinical cases, it is important to note that LA‐OMEP should only be used in dogs with a clear indication for gastric acid suppression, utilizing the guidelines established by the American College of Veterinary Internal Medicine Consensus Statement on the rational use of gastroprotectants.^16^ The PPIs and other gastroprotectants are often used inappropriately and overprescribed by general practitioners and veterinary specialists.^17^, ^18^, ^19^ The authors advise judicious use of this medication for dogs where severe GI bleeding, treatment compliance, or both is a concern.

There was individual variability in response to LA‐OMEP demonstrated by the dogs. The individual variation in the response to LA‐OMEP was further highlighted in the 4 dogs where pH data were available after day 7. In 1 of those dogs, gastric acid suppression met and exceeded human clinical goals on day 8. And while clinical goals were not met in the remaining 3 dogs after day 7, 2 of 3 still had evidence of gastric acid suppression on day 8. The remaining dog had pH values that had returned to near baseline by day 8. The reasons for this variability are unknown but have been observed in other gastric acid suppressant studies in dogs.^20^ The cause of the individual variation seen in the present study is still undetermined but was also observed in a study evaluating LA‐OMEP in horses.^12^ Proposed reasons for the variation in the present study include individual variation in drug metabolism between dogs, variations in the rate of inhibition of hydrogen‐potassium ATPase pumps at the site of action, variations in the accuracy of deposition of LA‐OMEP in the muscle or variation in the rate and extent of absorption of LA‐OMEP. Although the authors tried to ensure accurate injection of LA‐OMEP by standardizing injection techniques and limiting administration of the injection to 2 of the authors (G. Galyon and A. Odunayo), it is possible that some of the drug might have been inadvertently deposited subcutaneously, thus altering the pharmacokinetics of the drug. The LA‐OMEP is suspended in a vehicle specially selected for tissue tolerance and manufactured to ensure quick and reliable resuspension. However, this formulation is a suspension and some separation of the components occurs during storage. Despite due diligence to ensure that the drug was properly resuspended before administration to each dog, it is possible that there was variability in the actual amount of omeprazole administered, leading to the variation in response seen in the dogs. The effect of the change in intragastric pH after LA‐OMEP administration on gastric and esophageal healing in dogs remains to be investigated but is likely to be helpful in guiding decisions for redosing. The onset of action of LA‐OMEP was rapid, with a mean onset of 98.11 minutes, although it should be noted that this determination was based on the time the intragastric pH was sustained at a pH ≥4. Thus, LA‐OMEP confers the additional advantage of a rapid onset of action in critically ill dogs with profound GI bleeding.

The authors decided to use the epaxial muscle bed as the injection site due to ease of observing injection site reactions with minimal disruption of the dogs. However, 1 study demonstrated that the fastest absorption of dexmedetomidine and hydromorphone was achieved after injection into the semimembranosus muscle.^19^ It is difficult to say if absorption pharmacokinetics will be similar for LA‐OMEP, however an average onset of action of 98.11 minutes is still timely for most critically ill dogs with GI bleeding or moderate to severe esophagitis.

The LA‐OMEP dose used in the present study, 4 mg/kg IM as an extrapolation from the equine studies, appears to be an effective and safe dose in dogs. Additional studies evaluating higher or lower doses, in terms of duration of action and efficacy, might be considered in the future.

Rebound acid hypersecretion (RAH), a phenomenon in which hypergastrinemia caused by prolonged drug‐induced inhibition of gastric acid secretion results in acid hypersecretion after discontinuation of acid suppressant treatment, occurs in human patients with a history of extended use of acid suppressants and in cats after 60 days of omeprazole treatment.^21^, ^22^, ^23^ It is estimated that long‐term (usually 3 months or longer, although it could happen after 28 days of treatment) PPI treatment causes moderate hypergastrinemia and rebound hypersecretion in about 30% to 40% of human patients when PPIs are abruptly discontinued.^22^, ^24^, ^25^ This leads to symptoms of gastroesophageal reflux (heartburn, regurgitation, and burning sensation in the esophagus) in humans. Recommendations are made for humans to wean off PPIs in patients who have received them for an extended time or switch them to a less effective acid blocker (eg, histamine‐2 receptor antagonists).^22^ The incidence of RAH is unknown in dogs. In a study of dogs receiving 2 weeks of famotidine treatment, serum gastrin levels were increased after 3 days of famotidine administration but decreased in most of the dogs by day 12 of treatment.^26^ Similar results were found in another study evaluating gastrin levels in dogs treated with famotidine for 14 days.^27^ Although LA‐OMEP produced potent intragastric acid suppression in the dogs in the present study, on average, its effect decreased slowly over time. On average, the mean intragastric pH and MPT pH was still higher than baseline on day 7. Future studies are needed to evaluate how quickly the intragastric pH returns to baseline in dogs. However, RAH should be considered in dogs treated with LA‐OMEP with 1 or more doses, although the benefit with repeated dosing is currently undetermined.

The software tracing utilized in this study makes it easy to determine if the pH capsule is still in the stomach. Despite excellent intragastric acid suppression noted in the present study, when the capsule is located in the stomach, multiple acid spikes can be observed during the day with the intragastric pH approaching <4 for periods of time. When the capsule moves into the small intestine, the pH tracing stays persistently higher than pH 4 without acid spikes. This observation prompts an abdominal radiograph to investigate for capsule migration.

LA‐OMEP‐related adverse effects were minimal in the present study. All episodes of vomiting, hyporexia, and diarrhea were associated with the days the dogs were sedated for capsule placement. All adverse effects were resolved within 24 hours of sedation for all dogs. One dog had a seizure shortly after the LA‐OMEP injection. Diagnostic testing and a neurologic exam suggested idiopathic epilepsy, although an MRI or a CSF tap were not performed. Further investigation revealed a history of seizures in the lineage of that dog. Although the dog did not complete the study, no additional seizures occurred during the duration of the study. The dog was reported to have a single seizure about 3 months after the study was completed, which is more consistent with idiopathic epilepsy.

This study had an unusually high frequency of capsule failure during placement. The manufacturers had a recall of capsules because of similar issues and also sent out an urgent “Field Safety Alert” after the present study was completed, notifying users of similar feedback of a high capsule failure rate. Although the capsules used in the present study were not part of the recalled lot, it is likely that a defect in capsule manufacture led to the high failure rate. Although the Bravo pH technology allows data recording for 72 to 96 hours, the authors decided to download the data every 24 hours, in order to assess for early capsule migration to facilitate earlier replacement.

Limitations of the current study include the absence of pharmacokinetic and limited pharmacodynamic information, the limited number of dogs with pH information after day 7 and the exclusion of dogs with clinical GI bleeding or suspicion for GI disease. Additionally, a small amount of data was lost when early capsule migration occurred prior to capsule replacement. The technology records approximately 14 000 pH measurements per 24 hours and, on average, 274 measurements were lost per dog every 24 hours, with most pH being relatively stable across the data. It is unlikely that these lost data led to erroneous conclusions.

In summary, the LA‐OMEP formulation produced excellent gastric acid suppression in healthy dogs for an average of 5 days and up to 7 days, after a single intramuscular injection.

## CONFLICT OF INTEREST DECLARATION

Dr. M. Katherine Tolbert is a member of the TriviumVet scientific advisory board. No other authors have a conflict of interest.

## OFF‐LABEL ANTIMICROBIAL DECLARATION

Authors declare no off‐label use of antimicrobials.

## INSTITUTIONAL ANIMAL CARE AND USE COMMITTEE (IACUC) OR OTHER APPROVAL DECLARATION

The University of Tennessee, approval number 2814‐0221.

## HUMAN ETHICS APPROVAL DECLARATION

Authors declare human ethics approval was not needed for this study.

## References

[1] Waldrop JE , Rozanski EA , Freeman LM , Rush JE . Packed red blood cell transfusions in dogs with gastrointestinal hemorrhage: 55 cases (1999–2001). J Am Anim Hosp Assoc. 2003;39:523‐527. 14736715 10.5326/0390523

[2] Lynch A , Respess M , Boll A , et al. Hospital‐acquired anemia in critically ill dogs and cats: a multi‐institutional study. J Vet Intern Med. 2016;30:141‐146. 26578290 10.1111/jvim.13650 PMC4913629

[3] Stiller J , Defarges AM , Brisson BA , et al. Diagnostic evaluation of urea nitrogen/creatinine ratio in dogs with gastrointestinal bleeding. J Vet Intern Med. 2021;35:1427‐1438. 33728701 10.1111/jvim.16101 PMC8162593

[4] Stanley AJ , Laine L . Management of acute upper gastrointestinal bleeding. BMJ. 2019;364:l536. 30910853 10.1136/bmj.l536

[5] Tolbert MK , Odunayo A , Howell R , et al. Efficacy of intravenous administration of combined acid suppressants in healthy dogs. J Vet Intern Med. 2015;29:556‐560. 25711717 10.1111/jvim.12555 PMC4895496

[6] Sreedharan A , Martin J , Leontiadis GI , et al. Proton pump inhibitor treatment initiated prior to endoscopic diagnosis in upper gastrointestinal bleeding. Cochrane Database Syst Rev. 2010;7:CD005415. 10.1002/14651858.CD005415.pub3 PMC6769021 20614440

[7] Bell N , Burget D , Howden C , et al. Appropriate acid suppression for the management of gastro‐oesophageal reflux disease. Digestion. 1992;51:59‐67. 1397746 10.1159/000200917

[8] Burget DW , Chiverton SG , Hunt RH . Is there an optimal degree of acid suppression for healing of duodenal ulcers?: a model of the relationship between ulcer healing and acid suppression. Gastroenterology. 1990;99:345‐351. 2142113 10.1016/0016-5085(90)91015-x

[9] Bersenas AM , Mathews KA , Allen DG , et al. Effects of ranitidine, famotidine, pantoprazole, and omeprazole on intragastric pH in dogs. Am J Vet Res. 2005;66:425‐431. 15822586 10.2460/ajvr.2005.66.425

[10] Tolbert K , Bissett S , King A , et al. Efficacy of oral famotidine and 2 omeprazole formulations for the control of intragastric pH in dogs. J Vet Intern Med. 2011;25:47‐54. 21143305 10.1111/j.1939-1676.2010.0651.x

[11] Kuhl A , Odunayo A , Price J , et al. Comparative analysis of the effect of IV administered acid suppressants on gastric pH in dogs. J Vet Intern Med. 2020;34:678‐683. 32020689 10.1111/jvim.15718 PMC7096616

[12] Sykes B , Kathawala K , Song Y , et al. Preliminary investigations into a novel, long‐acting, injectable, intramuscular formulation of omeprazole in the horse. Equine Vet J. 2017;49:795‐801. 28397996 10.1111/evj.12688

[13] Gough S , Hallowell G , Rendle D . A study investigating the treatment of equine squamous gastric disease with long‐acting injectable or oral omeprazole. Vet Med Sci. 2020;6:235‐241. 31945806 10.1002/vms3.220 PMC7196684

[14] Lane MB , Larson JC , Stokes JE , Tolbert MK . Continuous radiotelemetric monitoring of intragastric pH in a dog with peptic ulceration. J Am Vet Med Assoc. 2017;250:530‐533. 28207313 10.2460/javma.250.5.530

[15] VCOG . Veterinary cooperative oncology group ‐ common terminology criteria for adverse events (VCOG‐CTCAE) following chemotherapy or biological antineoplastic therapy in dogs and cats v1.1. Vet Comp Oncol. 2011;14:417‐446. 10.1111/vco.283 28530307

[16] Marks SL , Kook PH , Papich MG , Tolbert MK , Willard MD . ACVIM consensus statement: support for rational administration of gastrointestinal protectants to dogs and cats. J Vet Intern Med. 2018;32:1823‐1840. 30378711 10.1111/jvim.15337 PMC6271318

[17] McCormack R , Olley L , Glanemann B , Swann JW . Prospective observational study of the use of omeprazole and maropitant citrate in veterinary specialist care. Sci Rep. 2020;10:1‐13. 32978503 10.1038/s41598-020-72950-3 PMC7519060

[18] Baptista R , Englar R , São Braz B , Leal R . Survey‐based analysis of current trends for prescribing gastrointestinal protectants among small‐animal general practitioners in Portugal. Vet Sci. 2021;8:70. 33922570 10.3390/vetsci8050070 PMC8146071

[19] Duxbury S , Sorah E , Andrews P , Tolbert MK . Evaluation of proton pump inhibitor use in canine patients hospitalized in a tertiary referral hospital. J Vet Intern Med. 2020;34:2952. 10.1111/jvim.16491 PMC9511098 35866265

[20] Gaier A , Price J , Grubb L , Fitzgerald S , Tolbert MK . A prospective, randomized, masked, placebo‐controlled crossover study for the effect of 10 mg omeprazole capsules on gastric pH in healthy dogs. J Vet Intern Med. 2021;35:887‐891. 33586200 10.1111/jvim.16061 PMC7995404

[21] Fossmark R , Johnsen G , Johanessen E , Waldum HL . Rebound acid hypersecretion after long‐term inhibition of gastric acid secretion. Aliment Pharmacol Ther. 2005;21:149‐154. 15679764 10.1111/j.1365-2036.2004.02271.x

[22] Stojanov DB , Koraćević G , Stojanov D , et al. Rebound phenomenon of proton pump inhibitor therapy. Acta Med Median. 2021;60:64‐68.

[23] Gould E , Clements C , Reed A , et al. A prospective, placebo‐controlled pilot evaluation of the effect of omeprazole on serum calcium, magnesium, cobalamin, gastrin concentrations, and bone in cats. J Vet Intern Med. 2016;30:779‐786. 27062346 10.1111/jvim.13932 PMC4913587

[24] Lødrup AB , Reimer C , Bytzer P . Systematic review: symptoms of rebound acid hypersecretion following proton pump inhibitor treatment. Scand J Gastroenterol. 2013;48:515‐522. 23311977 10.3109/00365521.2012.746395

[25] Niklasson A , Lindström L , Simrén M , et al. Dyspeptic symptom development after discontinuation of a proton pump inhibitor: a double‐blind placebo‐controlled trial. Am J Gastroenterol. 2010;105:1531‐1537. 20332770 10.1038/ajg.2010.81

[26] Parente NL , Bari Olivier N , Refsal KR , Johnson CA . Serum concentrations of gastrin after famotidine and omeprazole administration to dogs. J Vet Intern Med. 2014;28(5):1465‐1470. 25056694 10.1111/jvim.12408 PMC4895597

[27] Mordecai A , Sellon R , Mealey K . Normal dogs treated with famotidine for 14 days have only transient increases in serum gastrin concentrations. J Vet Intern Med. 2011;25:1248‐1252. 22092612 10.1111/j.1939-1676.2011.00826.x

